# Compact SchCas9 Recognizes the Simple NNGR PAM

**DOI:** 10.1002/advs.202104789

**Published:** 2021-12-06

**Authors:** Shuai Wang, Huilin Mao, Linghui Hou, Ziying Hu, Yao Wang, Tao Qi, Chen Tao, Yuan Yang, Chengdong Zhang, Miaomiao Li, Huihui Liu, Shijun Hu, Renjie Chai, Yongming Wang

**Affiliations:** ^1^ State Key Laboratory of Genetic Engineering School of Life Sciences Zhongshan Hospital Fudan University Shanghai 200438 China; ^2^ Experimental Center of Forestry in North China Chinese Academy of Forestry Beijing 102300 China; ^3^ Department of Cardiovascular Surgery of the First Affiliated Hospital & Institute for Cardiovascular Science Collaborative Innovation Center of Hematology State Key Laboratory of Radiation Medicine and Protection Medical College Soochow University Suzhou 215000 China; ^4^ State Key Laboratory of Bioelectronics School of Life Sciences and Technology Jiangsu Province High‐Tech Key Laboratory for Bio‐Medical Research Southeast University Nanjing 210096 China; ^5^ Co‐Innovation Center of Neuroregeneration Nantong University Nantong 226001 China; ^6^ Shanghai Engineering Research Center of Industrial Microorganisms Shanghai 200438 China

**Keywords:** CRISPR/Cas9, genome editing, PAM, SaCas9, SchCas9

## Abstract

Clustered regularly interspaced short palindromic repeat (CRISPR)/SaCas9 is the most popular tool for in vivo genome editing due to its high efficiency and small genome. The authors previously developed four SaCas9 orthologs as genome‐editing tools. Here, to expand the targeting scope, they investigate the diversity of protospacer adjacent motifs (PAMs) by screening a list of 16 SaCas9 orthologs, twelve of which display editing activity in mammalian cells. They recognize five types of PAMs: NNGRRT, NNGRRR, NNGRC, NNGA, and NNGR. Importantly, SchCas9 recognizes the simple NNGR PAM, representing the most relaxed PAM preference of compact Cas9s to date. It is further demonstrated that SchCas9 enables efficient genome editing in multiple human cell lines. Altogether, these compact Cas9 tools offer a new option for both basic research and clinical applications.

## Graphical abstract

The compact clustered regularly interspaced short palindromic repeat (CRISPR)/CRISPR‐associated protein‐9 (Cas9) system is a promising platform for therapeutic applications, but its targeting scope is largely restricted to the protospacer‐adjacent motif (PAM). To expand the targeting scope, a Cas9 (SchCas9) recognizing a simple NNGR (R = G or A) PAM is identified. SchCas9 enables efficient genome editing in multiple human cell lines.

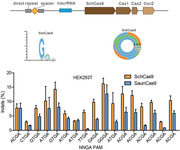

## 1 Introduction

The discovery of the RNA‐guided clustered regularly interspaced short palindromic repeat (CRISPR)/CRISPR‐associated protein (Cas) system has revolutionized the field of molecular biology and medicine.^[^ ^1^ ^]^ This system consists of a Cas nuclease and a guide RNA (gRNA) that directs Cas9 to cleave the target sites and generate double‐strand breaks (DSBs).^[^ ^1^ ^]^ The DSBs are repaired by either non‐homologous end joining (NHEJ) or homology‐directed repair (HDR) pathways, resulting in the desired mutations.^[^ ^2^ ^]^ Two types of Cas nucleases, including Cas9 ^[^ ^3^ ^]^ and Cas12,^[^ ^4^ ^]^ have been developed for genome editing. In addition to traditional genome editing, the CRISPR/Cas9 system can be used for base editing,^[^ ^5^ ^]^ prime editing,^[^ ^6^ ^]^ and gene activation/repression ^[^ ^7^ ^]^ by fusing catalytically‐impaired Cas9 protein to other enzymes. These new applications require the Cas9‐gRNA complex to precisely target genomic sites.

A key limitation to the application of CRISPR/Cas9 is the requirement for a specific protospacer adjacent motif (PAM) flanking the target. To address this limitation, one strategy is to engineer Cas9 nucleases to accept novel PAMs.^[^ ^8^, ^9^ ^]^ Another strategy is to harness natural Cas9 nucleases for genome editing,^[^ ^10^, ^11^, ^12^ ^]^ where each nuclease recognizes a defined PAM and a collection of these nucleases could cover all possible sequences. SpCas9 and its engineered variants can target nearly any sequence in the genome,^[^ ^9^, ^13^ ^]^ but these systems are too large to be delivered by a single adeno‐associated virus (AAV) for in vivo applications. Compact CRISPR/Cas9 systems can be delivered by a single AAV and hold great promise for therapeutic applications,^[^ ^10^, ^12^, ^14^ ^]^ but the targeting scope remains to be expanded.

Numerous CRISPR/Cas9 loci have been sequenced, but only a handful of them have been developed for genome editing. We recently developed four SaCas9 orthologs for mammalian genome editing,^[^ ^15^, ^16^ ^]^ expanding the targeting scope. In this study, to investigate the diversity of SaCas9 ortholog PAMs, we screened a list of 16 naturally occurring SaCas9 orthologs for genome editing. These Cas9 orthologs recognize multiple PAMs in mammalian cells. Interestingly, SchCas9 and its derivative, Sa‐SchCas9, recognize the simple NNGR PAM, representing the most relaxed PAM preference of compact Cas9s to date. Altogether, these compact CRISPR tools offer a new option for both basic research and clinical applications.

## 2 Results

### 2.1 Analysis of SaCas9 Orthologs

**Figure 1:**
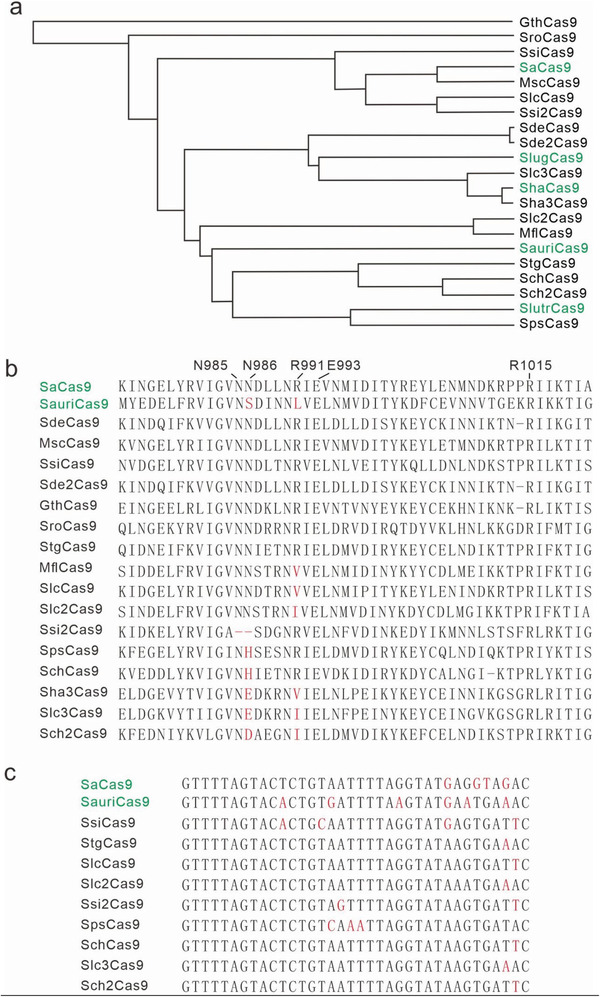
Analysis of SaCas9 orthologs. a) Phylogenetic tree of SaCas9 orthologs. Previously characterized Cas9 orthologs are shown in green. b) Amino acid sequence alignment of the SaCas9 ortholog PAM‐interacting (PI) domain. The residues that are important for PAM recognition are marked at the top; residues different from SaCas9 are highlighted in red. c) CRISPR direct repeat sequences alignment of SaCas9 orthologs.

We and others have demonstrated that genetically closely related Cas9 orthologs may recognize different PAMs.^[^ ^14^, ^15^, ^17^ ^]^ To investigate the diversity of SaCas9 ortholog PAMs, we used the SaCas9 sequence to search for related orthologs from NCBI's Gene database. We selected 16 SaCas9 orthologs with amino acid identity varying from 53.4% to 91.6% for characterization (**Figure** **1**a and **Table** **1**). A previous structural study revealed that five amino acids (N985, N986, R991, E993, and R1015) in the PAM‐interacting (PI) domain of SaCas9 are crucial for PAM recognition.^[^ ^18^ ^]^ Amino acid sequence alignment revealed that residues corresponding to N985, E993, and R1015 were highly conserved among these orthologs. In contrast, residues corresponding to N986 and R991 displayed substantial diversity. Eight of them differed in at least one residue among those corresponding to N986 and R991 (Figure 1b). One ortholog (Ssi2Cas9) contained two base pair deletions corresponding to N985 and N986. Seven orthologs contained identical residues corresponding to these five amino acids, allowing us to investigate whether they recognized the same PAM as SaCas9.

**Table 1:** Sixteen SaCas9 orthologs selected from NCBI database

NCBI ID	Host strain	Name	Length [aa]	Identity to SaCas9 [%]
WP_103167028	Staphylococcus devriesei	SdeCas9	1052	63.4
WP_096792116	Mammaliicoccus sciuri	MscCas9	1053	91.6
WP_105994700	Staphylococcus simulans	SsiCas9	1053	80.7
WP_083326835	Unclassified Staphylococcus	SlcCas9	1053	86.3
WP_050345681	Staphylococcus	Slc2Cas9	1058	62.3
WP_070855141	Staphylococcus sp. HMSC34C02	Slc3Cas9	1055	63.5
WP_145399953	Staphylococcus chromogenes	Sch2Cas9	1054	61.1
WP_103357343	Staphylococcus rostri	SroCas9	1052	58.2
WP_126496105	Mammaliicoccus fleurettii	MflCas9	1058	61.4
WP_107596301	Staphylococcus simulans	Ssi2Cas9	1051	83.1
WP_107506206	Staphylococcus devriesei	Sde2Cas9	1052	63.2
WP_107390356	Staphylococcus agnetis	StgCas9	1055	62.1
WP_104052030	Staphylococcus pseudintermedius	SpsCas9	1055	59.4
WP_153834605	Gracilibacillus thailandensis	GthCas9	1054	53.4
WP_107389582	Staphylococcus chromogenes	SchCas9	1054	61.8
WP_142813298	Staphylococcus haemolyticus	Sha3Cas9	1055	63.5

In addition to protein sequences, we also identified CRISPR direct repeat sequences and trans‐activating CRISPR RNA (tracrRNA) sequences for 9 Cas9 orthologs. Sequence alignment revealed that direct repeats and tracrRNAs were more conserved at the 5′ end than that at the 3′ end among SaCas9 orthologs (Figure 1c and Figure S1a, Supporting Information). We designed single guide RNA (sgRNA) scaffolds for each ortholog by fusing the 3′ end of a truncated direct repeat with the 5′ end of the corresponding tracrRNA, including the full‐length tail, via a 4‐nt linker (Figure S1b, Supporting Information). Interestingly, these sgRNAs formed similar secondary structures with three stem loops (Figure S2, Supporting Information), indicating that these SaCas9 orthologs could share the sgRNA scaffold for genome editing.

### 2.2 PAM Screening

**Figure 2:**
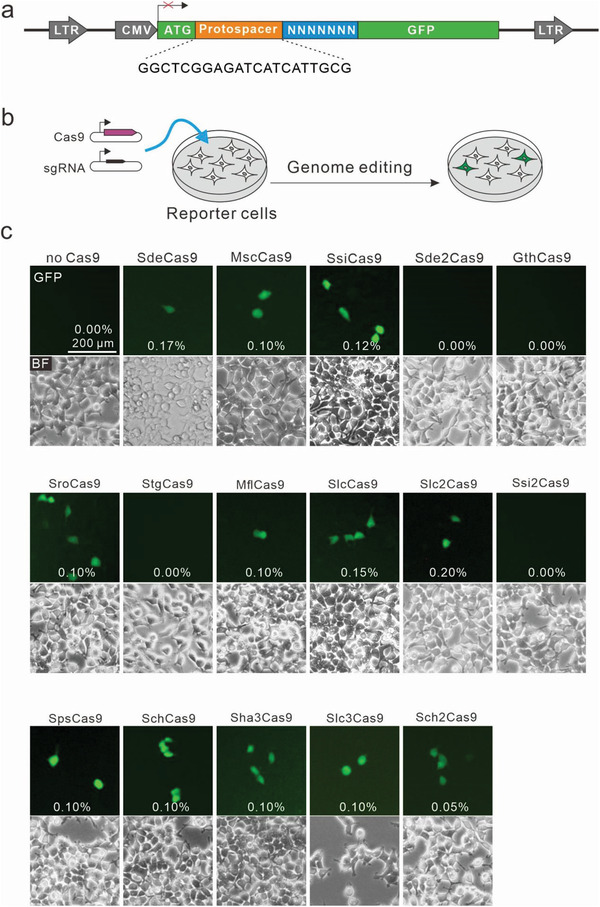
Screening of Cas9 ortholog activities with a GFP‐activation assay. a) Design of the GFP‐activation reporter construct. A target sequence (protospacer) followed by a 7‐bp random sequence is inserted between ATG and the GFP‐coding sequence. The library DNA is stably integrated into HEK293T cells. b) Procedure for Cas9 screening. The reporter cells are transfected with Cas9 and sgRNA expression plasmids. Genome editing restores the functional GFP cassette in a portion of the cells. c) Twelve out of 16 Cas9s could induce GFP expression. Percentage of GFP‐positive cells was shown.

Next, we used a previously established GFP‐activation assay for PAM screening.^[^ ^15^ ^]^ In this assay, a target sequence (protospacer) flanked by a 7‐bp random sequence is inserted into the GFP‐coding sequence immediately downstream of the ATG start codon, leading to a frameshift mutation. The reporter library is stably integrated into HEK293T cells. If a Cas9 nuclease has genome editing ability, it generates small insertions or deletions (indels) at the target sequence, and the functional GFP cassette will restore in a portion of the cells (**Figure** **2**a,b). Each Cas9 ortholog was human‐codon optimized, synthesized, and cloned into a mammalian SaCas9 expression plasmid that was developed by Ran F.A. et al.^[^ ^10^ ^]^ The canonical SaCas9 sgRNA scaffold was used for sgRNA expression.^[^ ^10^ ^]^ Three days after transfection of Cas9 with the sgRNA expression plasmids, GFP expression could be observed for 12 Cas9s (Figure 2c). Ssi2Cas9 showed no activity in mammalian cells, probably due to the deletion corresponding to SaCas9 N985 and N986.

**Figure 3:**
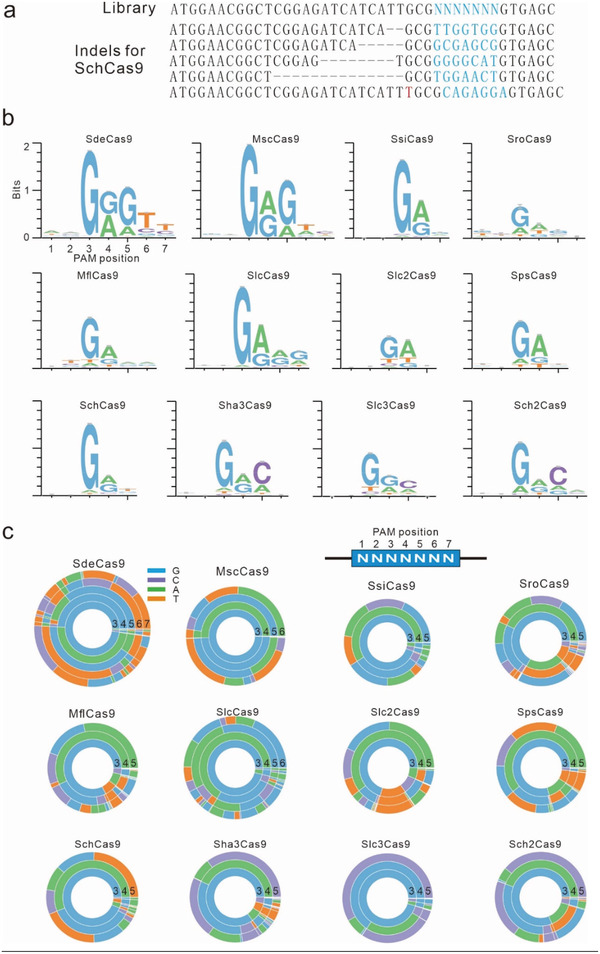
Analysis of the PAM sequence of Cas9. a) Examples of indels generated by SchCas9. b) WebLogos generated by analyzing the deep sequencing data. c) PAM wheels generated by analyzing the deep sequencing data.

We sorted GFP‐positive cells, and the target DNA was PCR‐amplified for deep sequencing. Sequencing results revealed that indels occurred at target sites (**Figure** **3**a). We generated WebLogos and PAM wheels based on deep‐sequencing data, which revealed that these Cas9s recognized diverse PAMs including NNGRRT (SdeCas9 and MscCas9), NNGRRR (SlcCas9), NNGRC (Sha3Cas9, Slc3Cas9, and Sch2Cas9), NNGR (SsiCas9, SroCas9, MflCas9, SpsCas9, and SchCas9), and NNGA (Slc2Cas9) (Figure 3b,c). Interestingly, although SsiCas9 and SroCas9 contained identical residues corresponding to SaCas9 N985, N986, R991, E993, and R1015, they recognized the simple NNGR PAM. These data further approved that genetically closely related Cas9 orthologs could recognize different PAMs.

### 2.3 Genome Editing With SchCas9

Next, we evaluated the capacity of these Cas9s for genome editing at a list of endogenous sites in HEK293T cells. We focused only on Cas9s with relatively simple PAMs including NNGR, NNGA, and NNGRC. Five days after transfection of Cas9 and sgRNA expression plasmids, genomic DNA was extracted, and target sites were PCR‐amplified. As an initial screen, we used the T7EI assay for rapid analysis of the efficiency for each Cas9. SchCas9 displayed higher efficiency than other Cas9s with the NNGR PAM (Figure S3a,b, Supporting Information). Sha3Cas9 displayed higher efficiency than other Cas9s with the NNGRC PAM. We chose these two Cas9s for further analysis.

**Figure 4:**
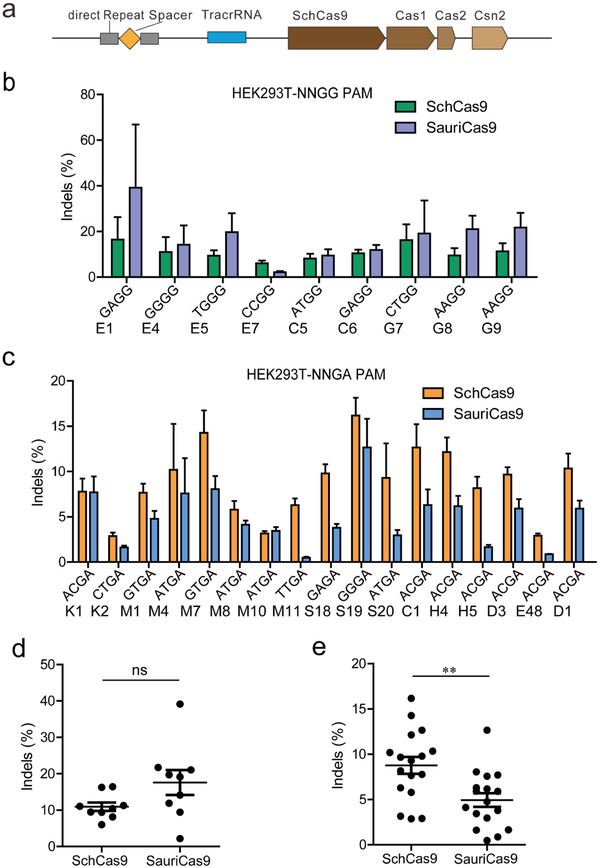
Genome editing capability of SchCas9. a) Schematic of the CRISPR/SchCas9 genetic locus. b) Genome editing with SchCas9 and SauriCas9 in a panel of 9 endogenous targets containing NNGG PAMs in HEK293T cells (mean ± SD, *n* = 3). c) Genome editing with SchCas9 and SauriCas9 in a panel of 17 endogenous targets containing NNGA PAMs in HEK293T cells (mean ± SD, *n* = 3). d) Quantification of indel efficiencies for Figure 4b (Student's *t*‐test, *n* = 9, *p < 0.05, **p < 0.01, ***p < 0.001). e) Quantification of indel efficiencies for Figure 4c (Student's *t*‐test, *n* = 17, *p < 0.05, **p < 0.01, ***p < 0.001).

The CRISPR/SchCas9 locus consists of four Cas genes, namely, Cas9, Cas1, Cas2, Csn2, 12 direct repeat sequences, and a putative tracrRNA (**Figure** **4**a). Protein sequence alignment revealed that SchCas9 (1054 amino acids) shared 61.8% sequence identity with SaCas9. SchCas9 enabled efficient generations of indels for both the NNGG and NNGA PAMs in HEK293T cells, as revealed by targeted deep sequencing data (Figure 4b,c). We previously developed a SauriCas9 which strongly preferred an NNGG PAM and moderately preferred an NNGA PAM.^[^ ^15^ ^]^ We compared the activities of SchCas9 and SauriCas9 side‐by‐side. Both Cas9s displayed comparable activities with NNGG PAMs, while SchCas9 displayed higher activities with NNGA PAMs (Figure 4b–e). SchCas9 could also generate indels in HeLa cells and mouse N2a cells with varying efficacies (Figure S4a,b, Supporting Information). To facilitate delivery efforts, we packaged SchCas9 together with its sgRNA into AAV2 and infected HEK293T, A375, HeLa, HCT116, and human foreskin fibroblast (HFF) cells. Indels were detected in all cell types, but the frequencies varied depending on the loci and cell types (Figure S5a–e, Supporting Information). These data demonstrated that SchCas9 offered a new option for mammalian genome editing.

Next, taking advantage of the small size and flexible PAM requirement, we tried to develop a SchCas9‐based cytosine base editor. SchCas9 nickase (SchCas9n) was generated by introducing a D9A mutation. The SpCas9 in BE4max ^[^ ^19^ ^]^ was replaced by SchCas9n to generate APOBEC1–SchCas9n–UGI (SchBE4max, Figure S6a, Supporting Information). To test the capability of SchBE4max, we used it to introduce human pathogenic mutations into cells. We selected six targets with NNGA PAMs that could not be edited by BE4max. KCNH2‐T474I ^[^ ^20^ ^]^ and KCNQ1‐Q530* ^[^ ^21^ ^]^ mutations induce long QT syndrome, while MYBPC3‐Q401*, MYBPC3‐Q1259*,^[^ ^22^ ^]^ MYH7‐R403W,^[^ ^23^ ^]^ and MYH7‐R719W ^[^ ^24^ ^]^ mutations induce hypertrophic cardiomyopathy. We transfected HEK293T cells with plasmids encoding SchBE4max and sgRNAs. After 7 days, targeted deep sequencing revealed that C‐to‐T base editing occurred with efficiencies over 30% (Figure S6b–g, Supporting Information). In addition to pathogenic mutations, other C‐to‐T conversions could be observed in the editing window, but they did not change amino acids.

### 2.4 A Chimeric Cas9 Nuclease for Efficient Genome Editing

**Figure 5:**
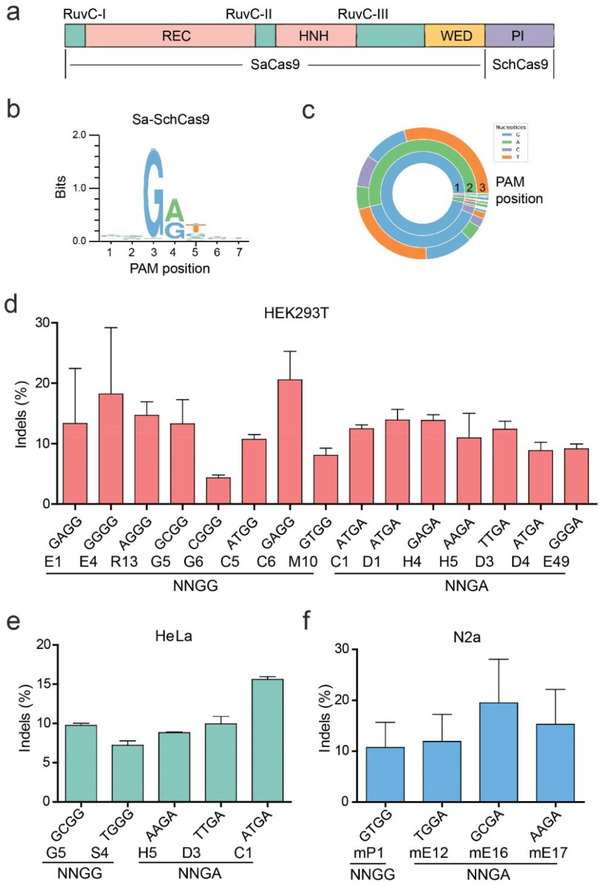
Characterization of chimeric Sa‐SchCas9 for genome editing. a) Schematic diagram of chimeric Sa‐SchCas9. b) WebLogo of Sa‐SchCas9 generated by analyzing the deep sequencing data. c) PAM wheel of Sa‐SchCas9 generated by analyzing the deep sequencing data. d–f) Sa‐SchCas9 generated indels at endogenous sites in HEK293T, HeLa and N2a cells (mean ± SD, *n* = 3).

We and others have demonstrated that the PI domain of closely related Cas9 orthologs can be exchanged, generating chimeric Cas9 nucleases with improved properties.^[^ ^14^, ^15^ ^]^ In this study, we used SchCas9 PI to replace SaCas9 PI, resulting in a chimeric Cas9 nuclease that we named “Sa‐SchCas9” (1055 aa, **Figure** **5**a). The GFP‐based PAM screening assay revealed that Sa‐SchCas9 preferred an NNGR PAM with a weak T preference at position 5 (Figure 5b,c). Sa‐SchCas9 enabled efficient editing in a panel of endogenous loci in HEK293T cells, HeLa cells, and mouse N2a cells (Figure 5d–f). The Sa‐SchCas9 and SchCas9 plasmids had the same structure, allowing us to compare their activities side‐by‐side (Figure S7a, Supporting Information). Western blot results revealed that the protein expression levels of these constructs were similar (Figure S7b, Supporting Information). Sa‐SchCas9 showed higher activity than SchCas9 on average for a panel of 9 loci in HEK293T cells (Figure S7c,d, Supporting Information). We packaged Sa‐SchCas9 together with its sgRNA into AAV2 and infected HEK293T, A375, HCT116, HeLa, and HFF cells. Indels were detected in all the cell types, but the frequencies varied depending on the loci and cell types (Figure S8a–e, Supporting Information). These data demonstrated that Sa‐SchCas9 was an alternative enzyme recognizing the NNGR PAM.

### 2.5 Specificity of SchCas9 and Sa‐SchCas9

Next, we evaluated the specificity of SchCas9 and Sa‐SchCas9 by using a previously developed GFP‐activation approach.^[^ ^15^ ^]^ SaCas9 was used as a control. This approach was similar to the PAM screening assay, but a fixed PAM was used (Figure S9a, Supporting Information). Once editing occurred, the GFP‐positive cells could be observed. The editing efficiency was reflected by the proportion of GFP‐positive cells. We generated a panel of sgRNAs with dinucleotide mutations along the protospacer. Individual Cas9 was transfected with a sgRNA into reporter cells and GFP expression was measured using flow fluorescence‐activated cell sorting (FACS). The results revealed that they tolerated mismatches at different positions (Figure S9a, Supporting Information). SaCas9 moderately tolerated mismatches at sgRNA positions 2 and 10 (counting the PAM as positions 22–25), and marginally tolerated mismatches at other positions. Sa‐SchCas9 moderately tolerated mismatches at a sgRNA position 6, and marginally tolerated mismatches at other positions. SchCas9 moderately tolerated mismatches at sgRNA position 3, and marginally tolerated mismatches at other positions. Overall, these three Cas9 nucleases displayed comparable specificity (Figure S9b, Supporting Information).

**Figure 6:**
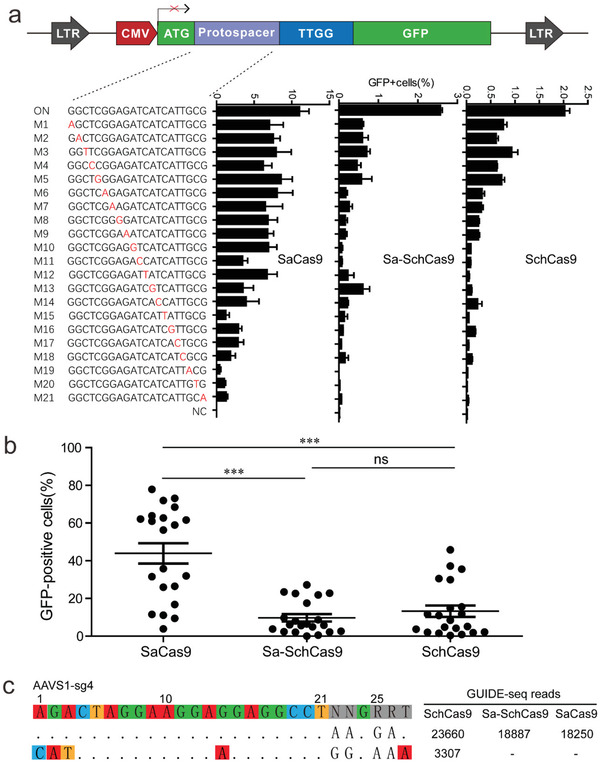
Analysis of the single mismatch tolerance of SchCas9, Sa‐SchCas9, and SaCas9. a) Schematic of the GFP‐activation construct is shown above. A panel of sgRNAs with a single nucleotide mutation (red) is shown below. The editing efficiency was calculated as the proportion of GFP‐positive cells (mean ± SD, *n* = 3). b) Comparison of editing efficiency at off‐target sites based on the GFP‐activation assay among SchCas9, Sa‐SchCas9, and SaCas9. The editing efficiencies at off‐target sites were normalized to the on‐target efficiency (ANOVA, *n* = 21, *p < 0.05, **p < 0.01, ***p < 0.001). c) GUIDE‐seq was performed to analyze the genome‐wide off‐target effects of SchCas9, Sa‐SchCas9, and SaCas9. On‐target and off‐target sequences are shown on the left. Read numbers are shown on the right. Mismatches compared to the on‐target site are shown and highlighted in color.

Next, we generated a panel of sgRNAs with a single nucleotide mutation along the protospacer to test the specificity of these Cas9s (**Figure** **6**a). SaCas9 tolerated a single mismatch at PAM‐distal positions 1–10 and 12, and moderately tolerated a single mismatch at other positions. Sa‐SchCas9 moderately tolerated a single mismatch at sgRNA positions 1–5 and 13, and less tolerated a single mismatch at sgRNA positions 6–12 and 14–21. SchCas9 moderately tolerated a single mismatch at sgRNA positions 1–9 and 14, and less tolerated a single mismatch at sgRNA positions 10–13 and 15–21. Overall, Sa‐SchCas9 and SchCas9 displayed higher specificity than SaCas9 (Figure 6b).

To analyze the genome‐wide off‐target effects of these three nucleases, GUIDE‐seq was performed.^[^ ^25^ ^]^ A target contained a PAM for all three Cas9 nucleases was selected (Figure 6c). Following transfection of the Cas9 plasmid, the sgRNA plasmid, and the GUIDE‐seq oligos into HEK293T cells for 5 days, we prepared libraries for deep sequencing. Sequencing analysis revealed that on‐target cleavage occurred for all the three Cas9 nucleases, as reflected by the high GUIDE‐seq read counts (Figure 6c). An off‐target site was identified for SchCas9, but no off‐target sites were identified for Sa‐SchCas9 and SaCas9, with this particular target.

SchCas9 recognizes a flexible PAM which may increase off‐target effects. We compared the specificity of SchCas9 and SauriCas9 by using GUIDE‐seq. We selected three targets (RUNX1_13, VEGFA_8, and FANCF_13) that have been used by Tan et al.,^[^ ^26^ ^]^ and two targets (EMX1_1 and EMX1_2) on the EMX1 gene. SauriCas9 induced more off‐targets than SchCas9 for each tested target. For example, SauriCas9 induced 22 off‐targets, while SchCas9 induced 14 off‐targets at VEGFA_8 locus (Figure S10a, Supporting Information). Of the five tested targets, SauriCas9 induced a total of 58 off‐targets, while SchCas9 induced a total of 28 off‐targets (Figures S10 and S11, Supporting Information). These data indicated that SchCas9 was more specific than SauriCas9. SauriCas9 mainly induced off‐targets with NNGG PAMs, while SchCas9 induced off‐targets with both NNGG and NNGA PAMs. Five SauriCas9‐induced off‐targets contained NNGA PAMs, while 13 SchCas9‐induced off‐targets contained NNGA PAMs. These data demonstrated that the flexible PAM contributed off‐target effects.

### 2.6 Genome Editing With Sha3Cas9

Protein sequence alignment revealed that Sha3Cas9 (1055 aa) shared 63.5% sequence identity with SaCas9. We selected a panel of 10 endogenous targets with the NNGRC PAM and tested Sha3Cas9 genome editing capacity. Sha3Cas9 enabled efficient editing for these targets in HEK293T cells, as revealed by targeted deep sequencing data (Figure S12a, Supporting Information). Next, we evaluated the specificity of Sha3Cas9 by using the GFP‐activation approach. We generated a panel of sgRNAs with dinucleotide mismatches along the protospacer. Sha3Cas9 displayed substantial off‐target activity with mismatched sgRNAs (M2‐M16, Figure S12b, Supporting Information). Therefore, further work was required to improve Sha3Cas9 specificity.

## 3 Discussion

Our study significantly expands the repertoire of the compact CRISPR toolbox. We demonstrated that 12 SaCas9 orthologs displayed activities in mammalian cells. Among these Cas9s, SchCas9 is particularly interesting because it enables efficient genome editing and recognizes the simple NNGR PAM, which occurs, on average, once in every ≈8 randomly chosen genomic locus. To our knowledge, this is the most relaxed PAM of compact Cas9s identified to date. We also generated a chimeric Sa‐SchCas9 that recognized the simple NNGR PAM and displayed high specificity. These constructs offer the opportunity to engineer genomic targets that were previously inaccessible by single AAV delivery. Due to their small size, these constructs may be used to treat genetic diseases, such as deafness, blindness, cardiomyopathy, and so on.

We previously identified four SaCas9 orthologs that recognize different PAMs, including NNGG, NNGRR, and NNGGV.^[^ ^15^, ^16^ ^]^ In this study, by screening a list of 16 SaCas9 orthologs, we identified Cas9s that recognize NNGRRT, NNGRRR, NNGRC, NNGA, and NNGR PAMs, respectively. These PAMs contain invariable G at position 3, G/A at position 4, and less conserved nucleotides at positions 5 and 6, which can be explained by the PI sequence of these Cas9s. These Cas9s contain a conserved residue corresponding to SaCas9 R1015 which forms bidentate hydrogen bonds with G at position 3, and a conserved residue corresponding to SaCas9 N985, which forms a water‐mediated hydrogen bond with G/A at position 4.^[^ ^18^ ^]^ These Cas9s contain diverse residue variations corresponding to SaCas9 N986 and R991 which play important roles in recognizing nucleotides at PAM positions 5 and 6.^[^ ^18^ ^]^ These rules are important for the identification of new genome editing tools in the future.

A major concern for utilizing CRISPR/Cas9 for gene therapy is the relatively high frequency of off‐target effects. The factors contributing to off‐target effects can be divided into two parts: tolerance of mismatches between guide RNA (gRNA) and DNA target and the PAM flexibility. The molecular mechanisms that control mismatch tolerance are not fully understood but multiple mechanisms may exist. Initially, researchers believed that the specificity could be improved by reducing the energetics of target site recognition, and developed eSpCas9(1.1) ^[^ ^27^ ^]^ and SpCas9‐HF1.^[^ ^28^ ^]^ Subsequently, Chen et al. demonstrated that a REC3 domain controlled Cas9 specificity.^[^ ^29^ ^]^ However, several amino acids outside of the REC3 domain have been shown to influence specificity.^[^ ^27^, ^28^ ^]^ Our studies revealed that the replacement of SaCas9 PI with SchCas9 PI improved specificity, suggesting that the PI domain contributed to specificity. We also experimentally proved that PAM flexibility influenced specificity. Cas9s with flexible PAMs facilitate targeting more genomic loci, while Cas9s with stringent PAMs can reduce off‐target effects. Researchers need to select an appropriate Cas9 from the CRISPR toolbox according to their applications.

## 4 Experimental Section

### Cell Culture and Transfection

HEK293T, HeLa, and HFF cells were cultured in DMEM (Gibco). N2a, HCT116, and A375 cells were cultured in 45% Opti‐MEM (Gibco) and 45% DMEM, McCoy's 5A medium (Gibco) or RPMI‐1640 medium (Gibco), respectively. All cell cultures were supplemented with 10% FBS (Gibco), and 1 × penicillin‐streptomycin (Gibco), and cells were grown at 37 °C with 5% CO_2_. HEK293T, HeLa, and N2a cells were transfected with Lipofectamine 2000 (Life Technologies) according to the manufacturer's instructions. For Cas9 PAM sequence screening, 1.2 × 10^7^ HEK293T cells were transfected with a total of 10 µg of Cas9 plasmid and 5 µg of sgRNA plasmid in 10‐cm dishes. For genome editing comparisons of Cas9, 10^5^ cells were transfected with a total of 300 ng of Cas9 plasmid and 200 ng of sgRNA plasmid in 48‐well plates.

### Plasmid Construction

Cas9 expression plasmid construction: the plasmid pX601 (addgene#61 591) was amplified by the primers px601‐F/px601‐R to obtain the pX601 backbone. The human codon‐optimized Cas9 gene (Table S1, Supporting Information) was synthesized by HuaGene (Shanghai, China) and cloned into the pX601 backbone by the NEBuilder assembly tool (NEB) according to the manufacturer's instructions. Sequences of each Cas9 were confirmed by Sanger sequencing (GENEWIZ, Suzhou, China).

sgRNA expression plasmid construction: the sgRNA expression plasmids were constructed by ligating sgRNA into the Bsa1‐digested hU6‐Sa_tracr plasmid. The primer sequences and target sequences are listed in Tables S2 and S3, Supporting Information, respectively.

### PAM Sequence Analysis

Twenty‐base‐pair sequences (AAGCCTTGTTTGCCACCATG/GTGAGCAAGG GCGAGGAGCT) flanking the target sequence (GAACGGCTCGGAGATCATC ATTGCGNNNNNNN) were used to fix the target sequences. GCG and GTGAGCAAGGGCG AGGAGCT were used to fix a 7‐bp random sequence. Target sequences with in‐frame mutations were used for PAM analysis. The 7‐bp random sequence was extracted and visualized by WebLogo^[^ ^30^ ^]^ and a PAM wheel chart to identify PAMs.^[^ ^31^ ^]^

### Genome Editing for Endogenous Sites

HEK293T, HeLa, and N2a cells were seeded into 48‐well plates and transfected with a total of 300 ng of Cas9 plasmid and 200 ng of sgRNA plasmid by Lipofectamine 2000 (1 µL). Cells were collected 5 days after transfection. Genomic DNA was isolated, and the target sites were PCR amplified and extracted by QuickExtract DNA Extraction Solution (Epicentre) for deep sequencing. For genomic HEK293T DNA, the PCR products were subjected to a T7E1 assay to check the editing efficiency. The primer sequences are listed in Table S2, Supporting Information.

### Test of Cas9 Specificity

To test the specificity of SchCas9, Sa‐SchCas9, and Sha3Cas9, we generated two GFP reporter cell lines with TTGG and GTGGC PAMs. The cells were seeded into 48‐well plates and transfected with 300 ng of Cas9 plasmids and 200 ng of sgRNA plasmids by using Lipofectamine 2000. Five days after editing, the GFP‐positive cells were analyzed on a Calibur instrument (BD). The data were analyzed using FlowJo.

### AAV Production

One day before transfection, ≈10^7^ HEK293T cells were seeded in a 10‐cm dish. For each well, 4 µg of Cas9‐sgRNA expressing plasmid, 4 µg of pAAV‐RC (Gene‐Bank: AF369963), and 8 µg of pAAV‐helper were transfected using 160 µL of PEI (0.1% m/v, Poly‐sciences, Cat# 23 966 [pH 4.5]). The medium was replaced with fresh DMEM after 8–10 h. After 72 h, the cells were scraped and poured into a new tube. The cells were centrifuged at 4000 rpm and 4 °C for 10 min, and the supernatant was transferred into a new 15‐mL tube. The cell pellet was resuspended in 1 mL of PBS and transferred to a new 1.5‐mL conical tube. Then, the cells were frozen in liquid nitrogen for 3–4 min and thawed at 37 °C for 5–10 min; this process was repeated 4 times. Then, the cells were centrifuged at 4000 rpm and 4 °C for 10 min. The two supernatants were mixed and filtered with a 0.45‐µm polyvinylidene fluoride filter. One‐half volume of the mixed solution was added and incubated at 4 °C overnight. After centrifugation at 4 °C for 2 h at 12 000 rpm, the flow‐through was discarded, and 500 µL of chilled RB TMS was added. Quantitative PCR revealed that the AAV titer was 2.0 × 10^9^ copies/mL. Twenty‐five microliters of the virus was added to a 48‐well plate at 60–80% cell confluency, resulting in a multiplicity of infection (MOI) of 10^3^ viral genomes/cell.

### GUIDE‐Seq

GUIDE‐seq experiments were performed as described previously,^[^ ^25^ ^]^ with minor modifications. Briefly, 2 × 10^5^ HEK293T cells were transfected with 500 ng of SchCas9/Sa‐SchCas9, 500 ng of sgRNA plasmids, and 100 pmol of annealed GUIDE‐seq oligonucleotides by electroporation and then seeded into 6 wells. The electroporation voltage, width, and the number of pulses were 1150 V, 30 ms, and 1 pulse, respectively. Genomic DNA was extracted with the DNeasy Blood and Tissue kit (QIAGEN) 6 days after transfection according to the manufacturer's protocol. The genome library was prepared and subjected to deep sequencing.^[^ ^25^ ^]^

### Western Blotting

One day before transfection, HEK293T cells were seeded into a 6‐well plate. For each well, 2 µg of Cas9‐expressing plasmid were transfected using 4 µL of Lipofectamine2000. Three days after transfection, cell samples were collected and total proteins were extracted using NP‐40 buffer (Beyotime) supplemented with 1 mm phenylmethanesulfonyl fluoride (PMSF) (Beyotime). The protein was separated by SDS‐PAGE gel and transferred onto polyvinylidene fluoride (PVDF) (Thermo) membrane. After transfer, the membrane was blocked with 5% (wt./vol.) BSA (Sigma) in TBS‐T (0.1% Tween 20 in 1 × TBS) buffer and then incubated in the primary antibody Anti‐HA tag (1:1000; ab236632, Abcam) and anti‐GAPDH (1:2000; 5174s, Cell Signaling) at 4 °C overnight. Wash membrane three times in TBS‐T for 5 min each time. The second antibody (1:10 000; ab6721, Abcam) was incubated for 1 h at room temperature, and then washed three times and imaged.

### Statistical Analysis

All the data are shown as mean ± SD. Statistical analyses were performed using Microsoft Excel. Two‐tailed, paired Student's *t*‐tests were used to determine statistical significance when comparing two groups, whereas analyses of variance (ANOVA) are used for comparisons between for three or more groups. A value of *p* < 0.05 was considered to be statistically significant (*p < 0.05, **p < 0.01, ***p < 0.001).

## Conflict of Interest

Authors applied a patent related to the work.

## Supporting information

Supporting Information

Supporting Table 1

Supporting Table 2

Supporting Table 3

Supporting Information

## Data Availability

The data that support the findings of this study are available in the supplementary material of this article.
