# Supplementary material for: Compact SchCas9 Recognizes the Simple NNGR PAM
Source: Adv Sci (Weinh). 2021 Dec 6;9(4):2104789. doi: 10.1002/advs.202104789 (PMC8811835; doi:10.1002/advs.202104789)
Supplement: Supplementary file 1 — Supporting Information [file ADVS-9-2104789-s001.pdf]

ADVANCED SCIENCE
Open Access

Supporting Information

for *Adv. Sci.,* DOI: 10.1002/advs.202104789

Compact SchCas9 Recognizes the Simple NNGR PAM

Shuai Wang, Huilin Mao, Linghui Hou, Ziying Hu, Yao Wang, Tao Qi, Chen Tao, Yuan Yang, Chengdong Zhang, Miaomiao Li, Huihui Liu, Shijun Hu, Renjie Chai, Yongming Wang

Compact SchCas9 recognizes the simple NNGR PAM

Shuai Wang, Huilin Mao, Linghui Hou, Ziying Hu, Yao Wang, Tao Qi, Chen Tao, Yuan Yang, Chengdong Zhang, Miaomiao Li, Huihui Liu, Shijun Hu, Renjie Chai, Yongming Wang

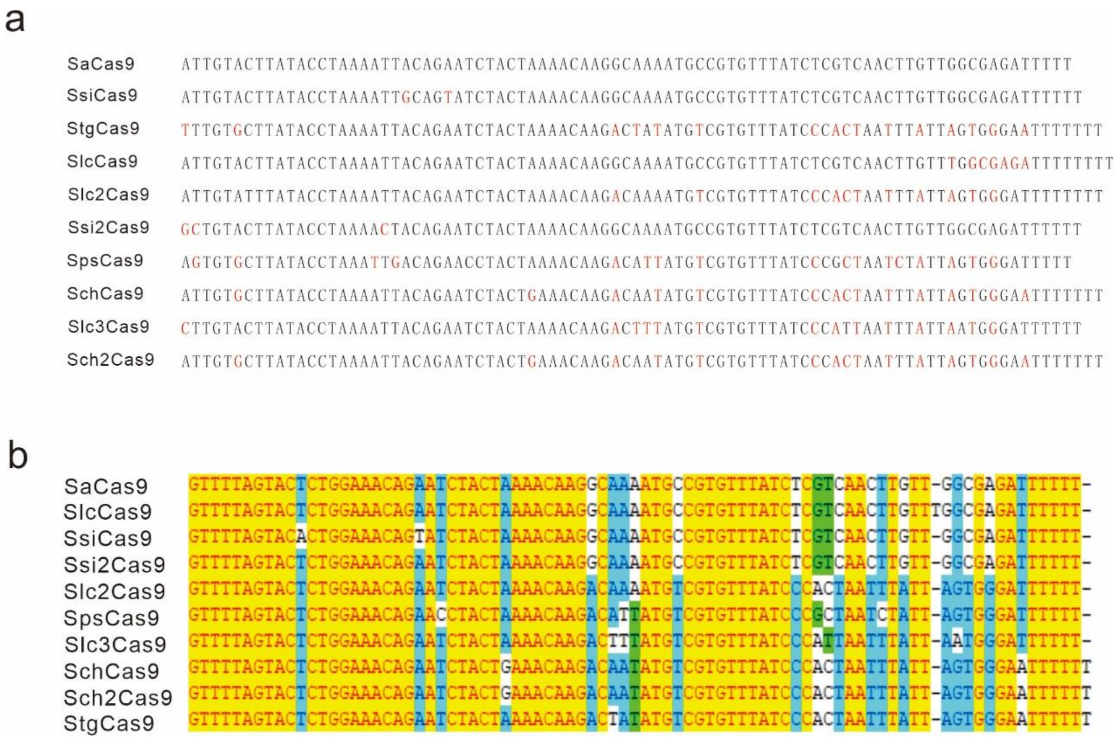

Figure S1. Analysis of tracrRNAs and sgRNAs. a) Alignment of tracrRNA for SaCas9 orthologs. b) Alignment of sgRNA scaffold for SaCas9 orthologs.

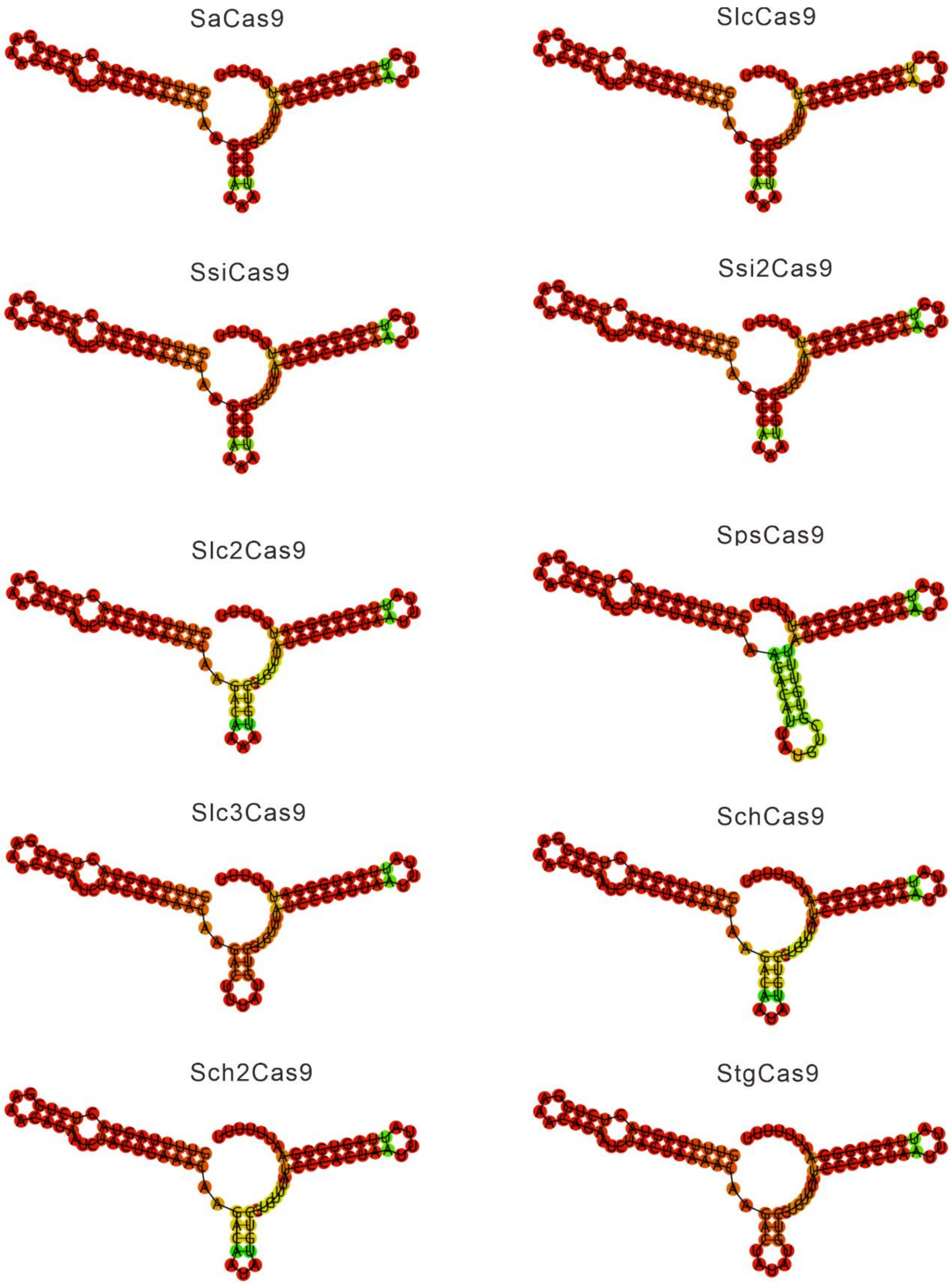

Figure S2. Analysis of SaCas9 orthologs' secondary RNA structures. These structures were generated by an online tool named RNAfold WebServer (http://rna.tbi.univie.ac.at/cgi-bin/RNAWebSuite/RNAfold.cgi).

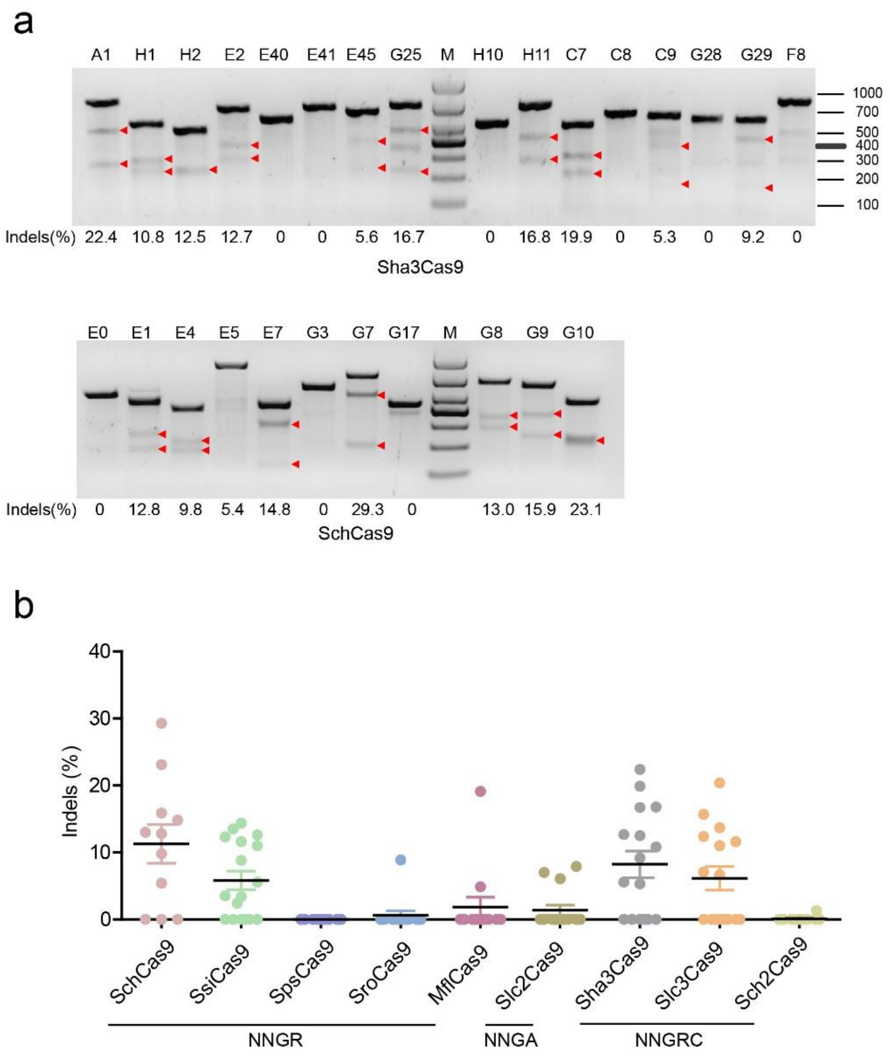

Figure S3. Evaluation of the genome editing efficiency of Cas9s. a) Examples of the gel pictures of T7EI assay. Cleaved fragments are marked by red triangles. Indel frequencies are shown below. b) Quantification of editing efficiency for 9 Cas9s.

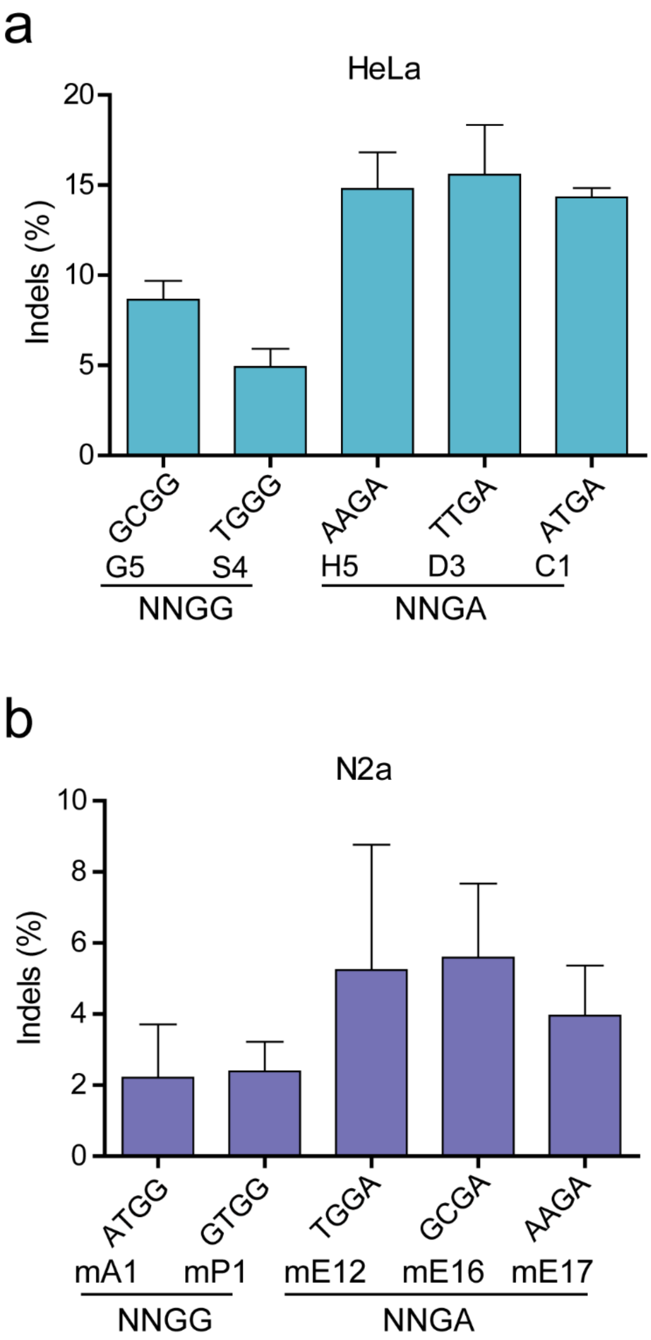

Figure S4. SchCas9 enables genome editing in HeLa and N2a cells. a) SchCas9 generated indels in 5 endogenous loci in HeLa cells (mean±SD, n=3). b) SchCas9 generated indels in 5 endogenous loci in N2a cells (mean±SD, n=3).

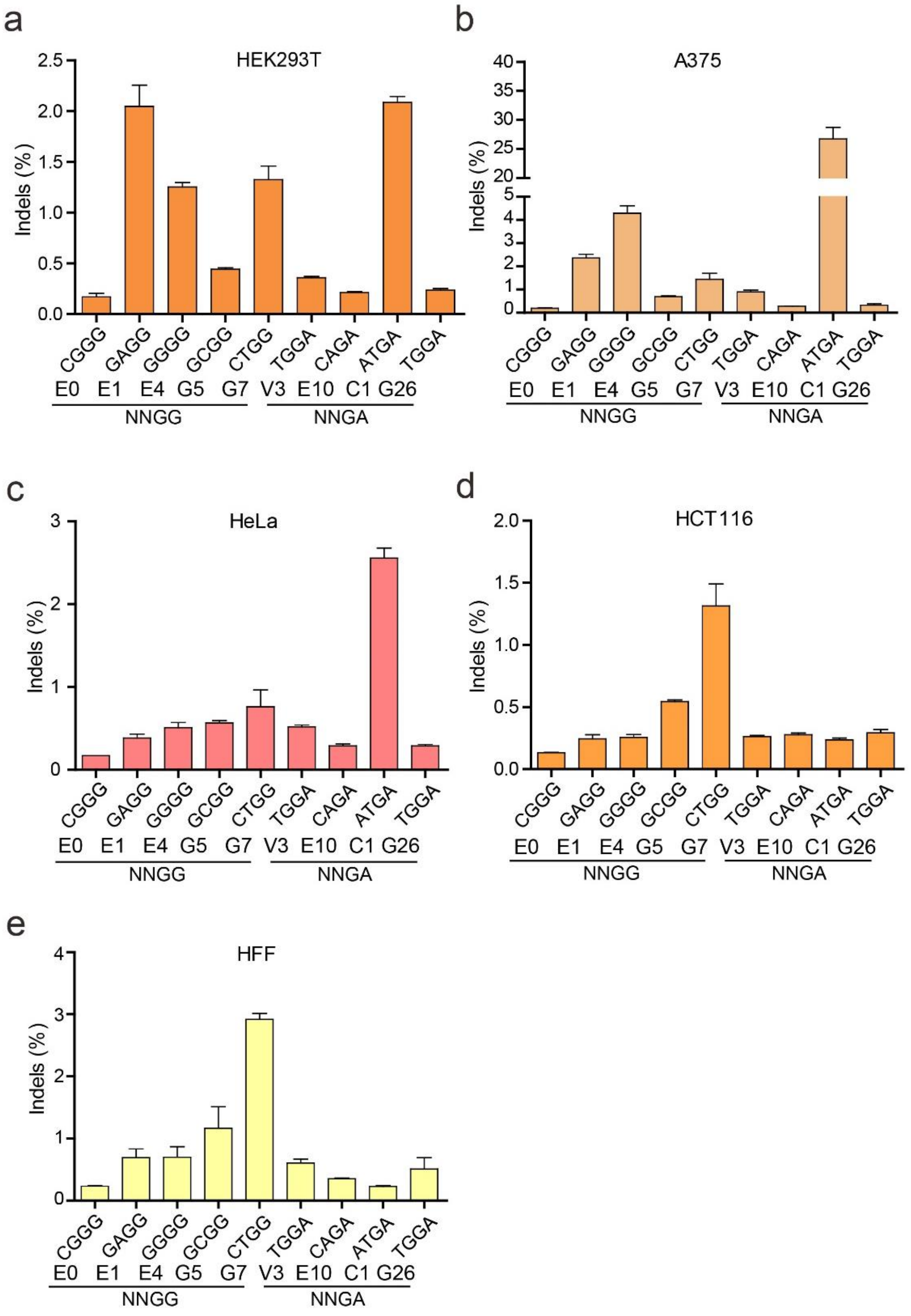

Figure S5. SchCas9 can be delivered by AAV for genome editing in a) HEK293T, b) A375, c) HeLa, d) HCT116 and e) human foreskin fibroblast (HFF) cells (mean±SD, n=3).

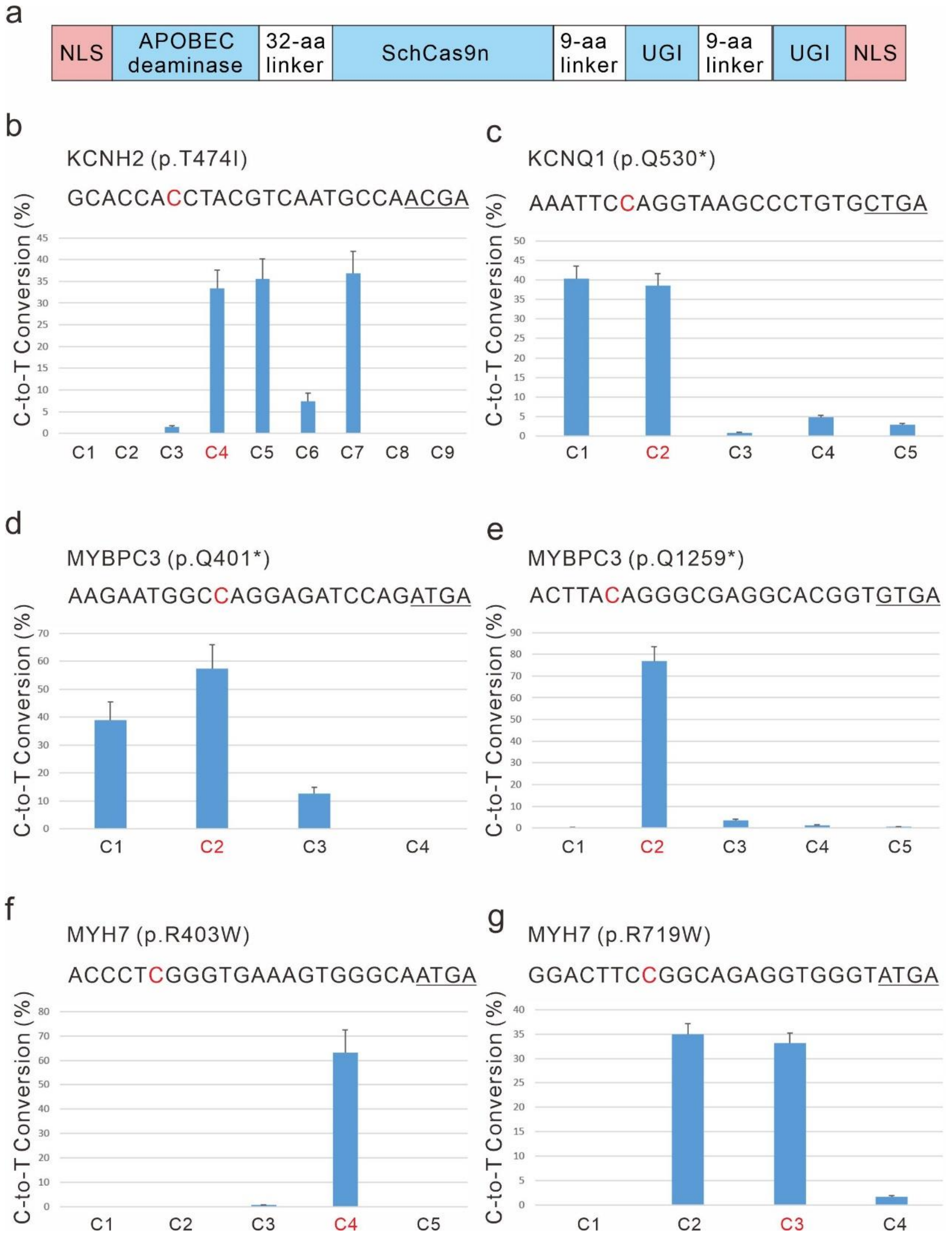

Figure S6. SchBE4max enables the introduction of pathogenic mutations into cells. a) Schematic of the SchBE4max construct. b-g) Base editing for a panel of 6 pathogenic mutations, including KCNH2-T474I, KCNQ1-Q530*, MYBPC3-Q401*, MYBPC3-Q1259*, MYH7-R403W and MYH7-R719W (mean ± SD, n=3). Target sequences are shown above. PAMs are underlined. Pathogenic mutations are shown in red.

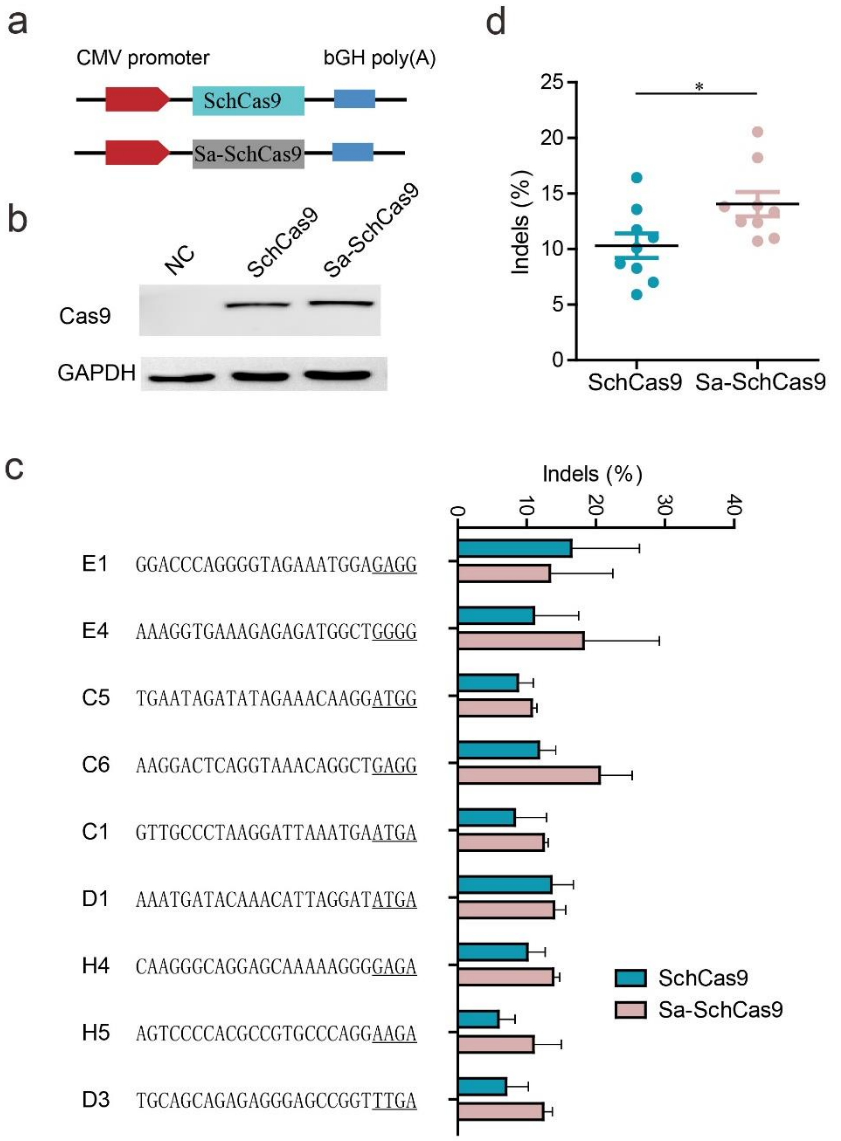

Figure S7. Genome editing with SchCas9 and Sa-SchCas9 for a panel of 9 endogenous loci. a) Schematic of SchCas9 and Sa-SchCas9 expression constructs. b) Western blot was used to measure the protein expression levels of SchCas9 and Sa-SchCas9. c) Genome editing with SchCas9 and Sa-SchCas9 for 9 endogenous loci (mean±SD, n=3). d) Comparison of the editing efficiency of SchCas9 and Sa-SchCas9 at 9 endogenous loci (Student's t-test, n=9, *$P \leq 0.05$).

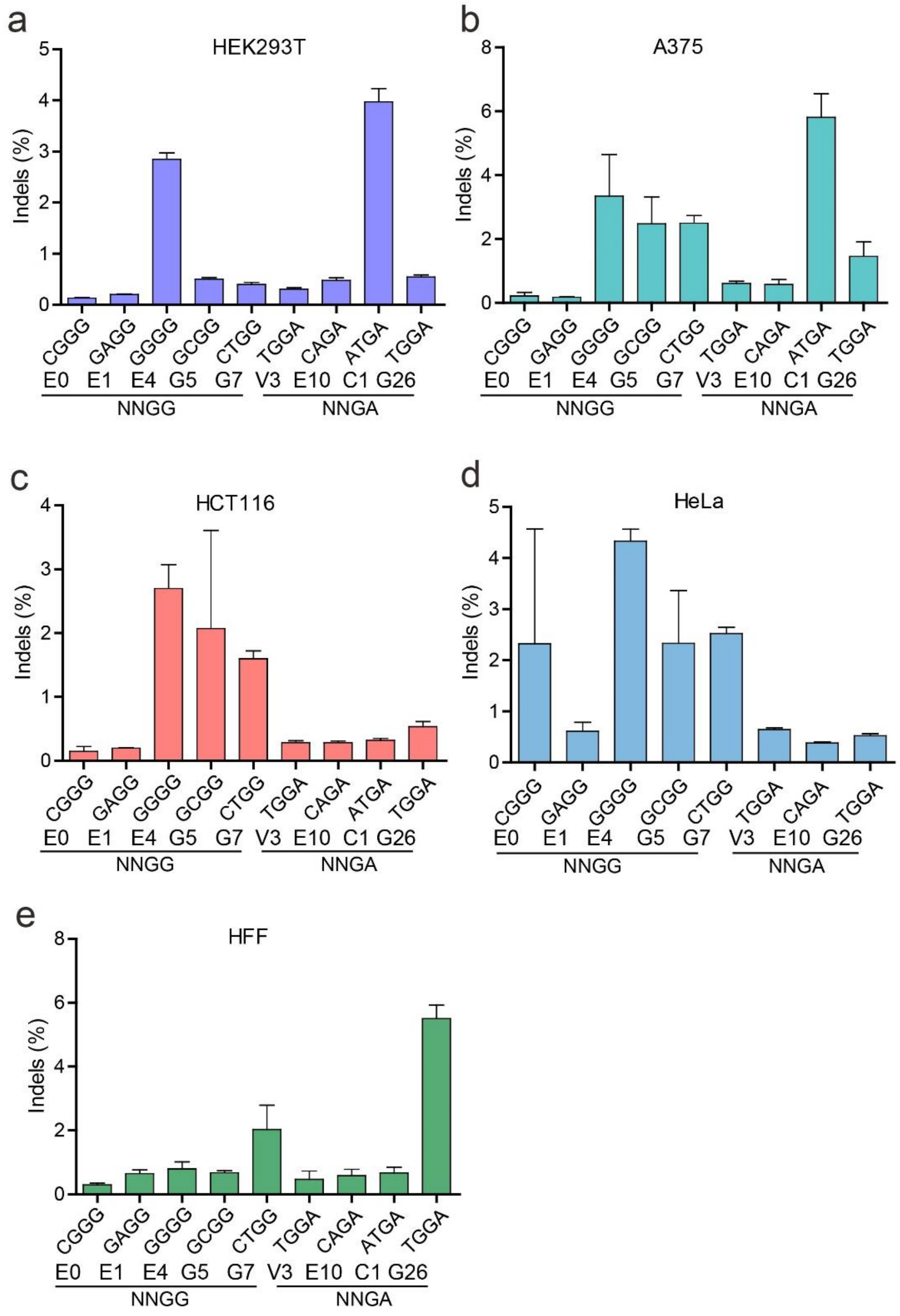

Figure S8. Sa-SchCas9 can be delivered by AAV for genome editing in a) HEK293T, b) A375, c) HeLa, d) HCT116 and e) human foreskin fibroblast (HFF) cells (mean±SD, n=3).

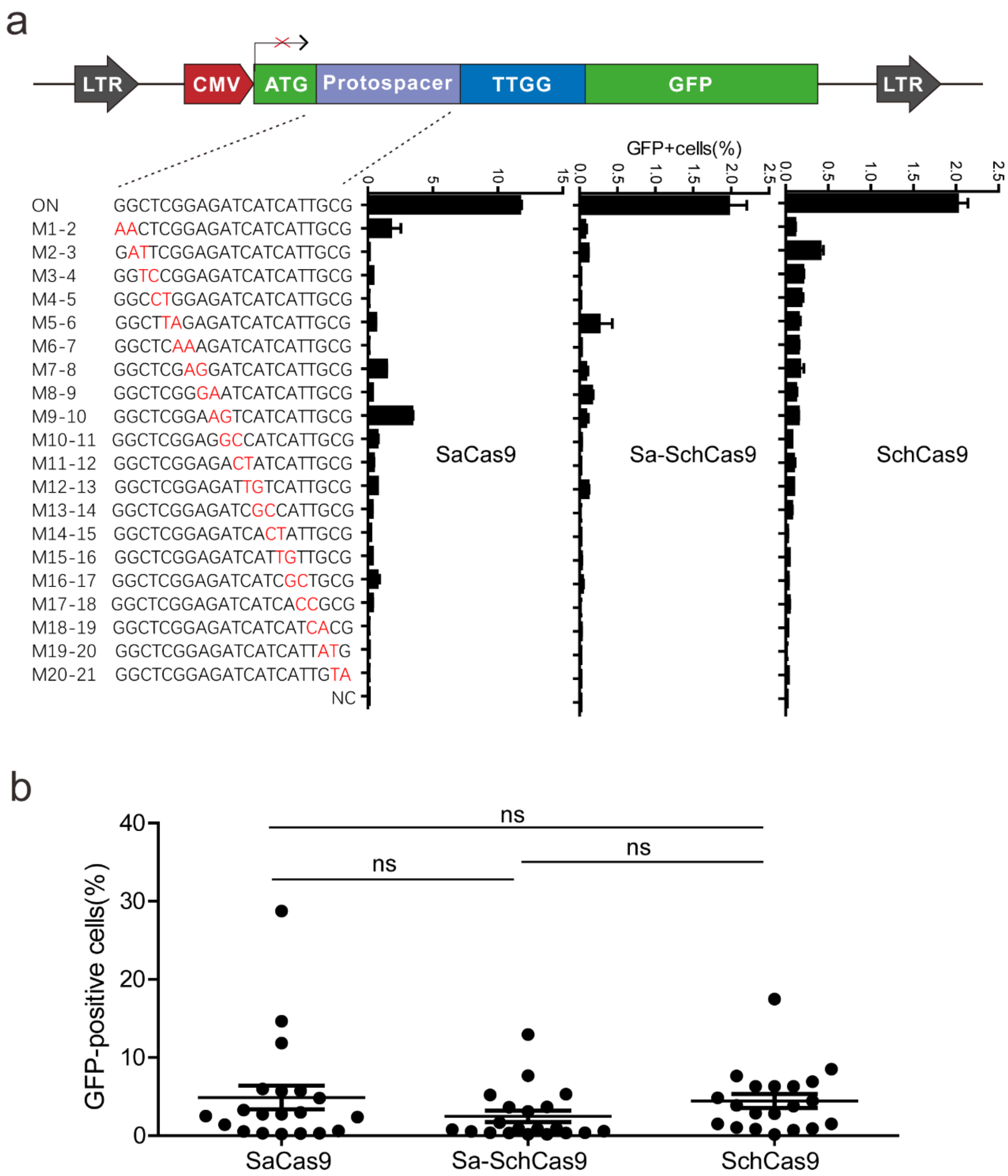

Figure S9. Analysis of the dinucleotide mismatch tolerance of SchCas9, Sa-SchCas9 and SaCas9. a) Schematic of the GFP-activation construct is shown above. A panel of sgRNAs with dinucleotide mutations (red) are shown below. The editing efficiency was calculated as the proportion of GFP-positive cells (mean ± SD, n=3). SaCas9 data was generated in a previous study [15]. b) Comparison of editing efficiency at off-target sites based on the GFP-activation assay among SchCas9, Sa-SchCas9 and SaCas9. The editing efficiencies at off-target sites were normalized to the on-target efficiency. ns: not significant. (ANOVA, n=20, *P < 0.05, **P < 0.01, ***P < 0.001).

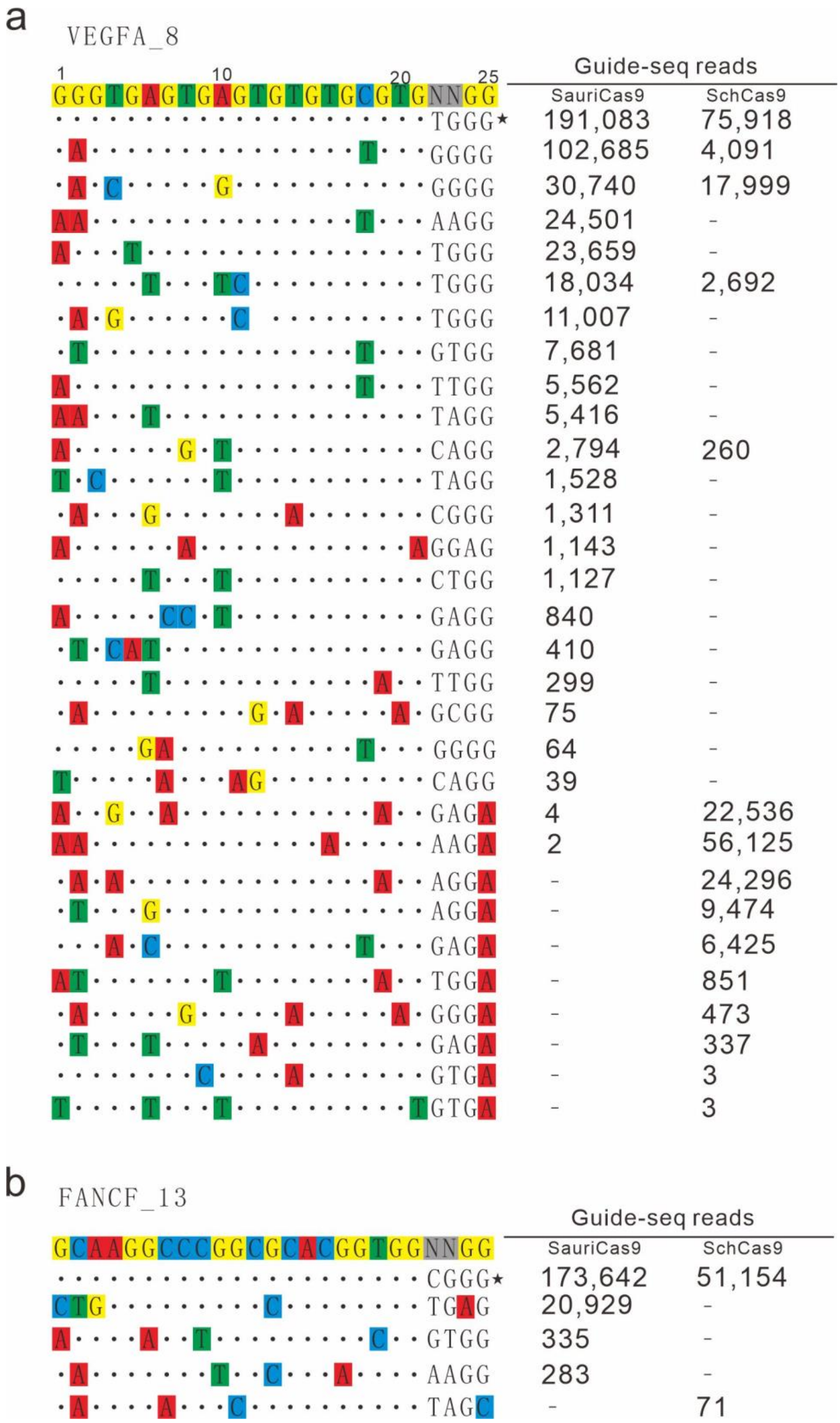

Figure S10. Analysis of genome-wide off-target effects for targets VEGFA_8 and FANCF_13. GUIDE-seq was performed to analyze the genome-wide off-target effects of SchCas9 and SauriCas9. On-target and off-target sequences are shown on the left. Read numbers are shown on the right. Mismatches compared to the on-target site are shown and highlighted in colour.

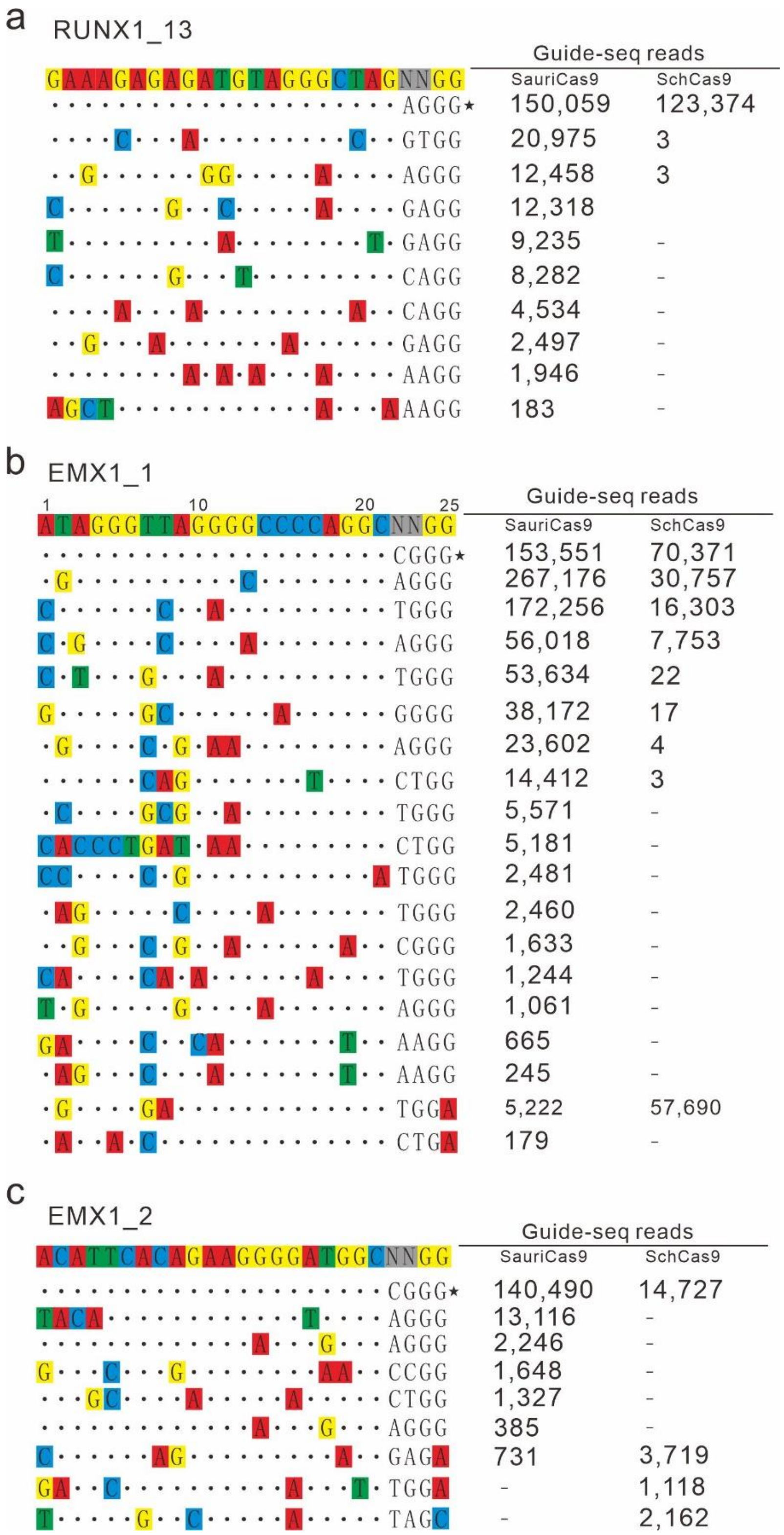

Figure S11. Analysis of genome-wide off-target effects for targets RUNX1_13, EMX1_1 and EMX1_2. GUIDE-seq was performed to analyze the genome-wide off-target effects of SchCas9 and SauriCas9. On-target and off-target sequences are shown on the left. Read numbers are shown on the right. Mismatches compared to the on-target site are shown and highlighted in colour.

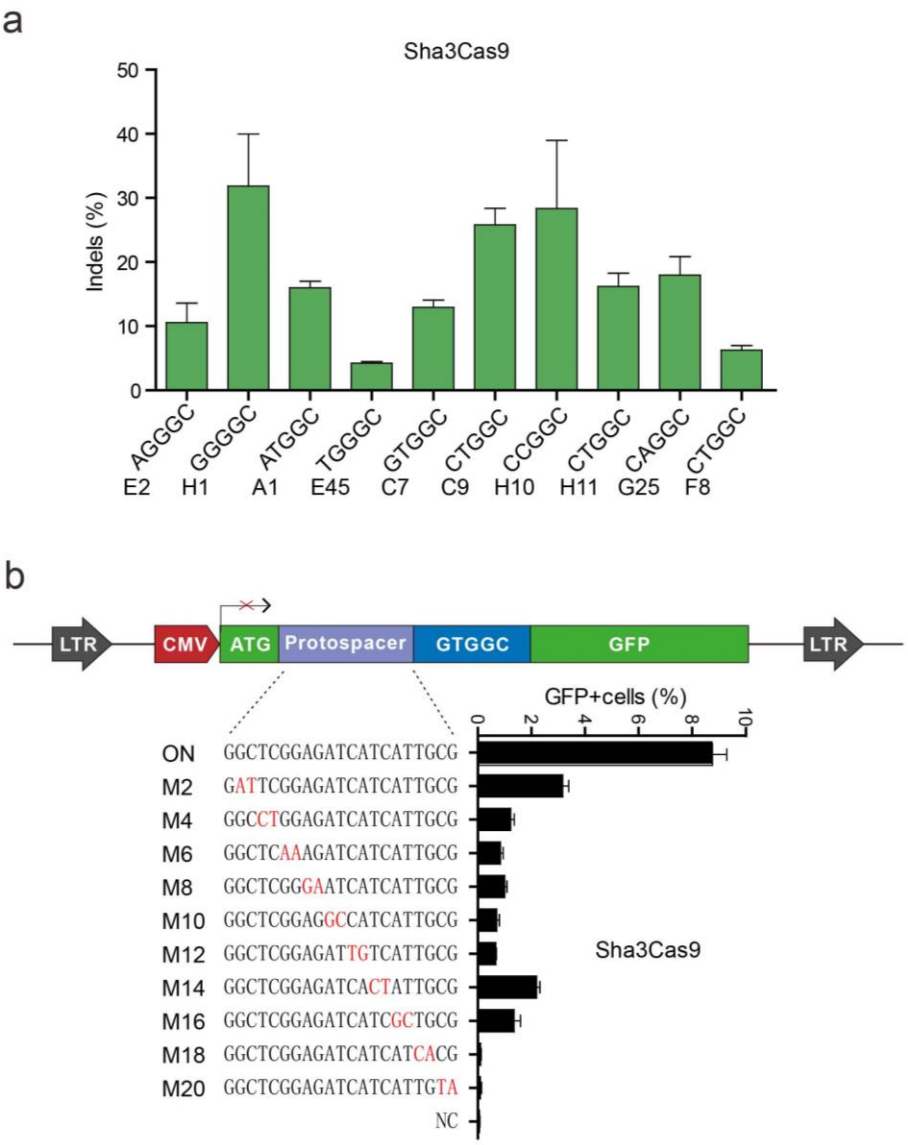

Figure S12. Genome editing capability of Sha3Cas9. (A) Genome editing with Sha3Cas9 for 13 endogenous loci in HEK293T cells (mean±SD, n=3). (B) Schematic of GFP-activation assay for specificity evaluation. A panel of sgRNAs with dinucleotide mutations (red) are shown below. The editing efficiency can be reflected by the ratio of GFP-positive cells (mean±SD, n=3).
